# Notes and News

**Published:** 1896-09-19

**Authors:** 


### NOTES AND NEWS.

(Collected and Communicated.)

The Bordeaux Assistance Publique has received a bequest of fr.160,000 from Madame Edmond Léon.

The Cape Town Station Hospital has been sold, its work being undertaken by the Wyneberg Hospital, which is to be enlarged.

Twenty cases of Berri-Beri have been reported at the Richmond Lunatic Asylum, Dublin. The disease so far has been of a mild form.

It is proposed to erect a monument to the memory of Pasteur at Munich. In Paris a part of the present Boulevard Vaugirard is henceforth to be known as the Boulevard Pasteur.

It is reported that the Salvation Army is about to establish an ambulance service, to be employed in removing drunkards from the streets and detaining them in shelters until restored to their normal condition.

Dr. Kingsbury has published an apology to Dr. A. G. Bateman, in which he withdraws the imputations he casts upon Dr. Bateman's honour and integrity in his pamphlet entitled "Medical Ethics." Dr. Kingsbury states that the imputation was unintentional.

The hospital ship *Victoria*, belonging to the Mission to Deep Sea Fishermen, is creating a great deal of interest at Hull, where she is now on exhibition. A large sale of work was held on board for the benefit of the mission. The ship was built in the Jubilee year.

The Nicolson-Mackenzie Memorial Hospital was opened at Strathpeffer last month by the Countess of Cromartie. The institution is intended for patients of the poorer classes, to enable them to receive the benefits of the waters. The institution has been established mainly through the generosity of the late Mrs. Morison Duncan. It is a pretty little building, built on a site presented by the late Lord Cromartie. It contains ten beds, and is under the charge of a trained nurse.

Dr. Yersin, the French doctor who in 1894 laboured so energetically during the outbreak of the plague in Hong Kong, claims that he has discovered a serum which cures the disease. He is carrying on his experiments in the seclusion of the French Missionary Seminary in Canton. His reported cures number over twenty.

The Austrian Alpine Club is about to establish a new order of succour called the Green Cross. It is to devote its labours to assisting Alpine climbers in distress or suffering from accidents. Châlets are to be established at various points of vantage containing suitable appliances, whilst Alpine guides are to receive instruction in applying simple remedies and in meeting emergencies.

The pressure during the holiday months is now so great that all inviting quarters are terribly overcrowded, and any hints as to new fields are welcome. We learn in Mrs. Ernest Hart's paper *Erin* that the Irish Broads possess much the same charms as those of the Mother Country. This is little known, to English people at any rate, and as the Norfolk Broads are now quite an expensive luxury, those with but moderately filled purses will be glad to hear that touring amongst the Irish Broads is remarkably cheap. The waters are more open than those of Norfolk, and the sailing is said to be splendid.

The new Samaritan Hospital in Glasgow was opened by Lady Bell last week. The foundation-stone was laid in May, 1895, and a complete description of the building, together with plans, was published in The Hospital for May 23rd, page 130. Until now the work has been carried on in premises in St. James' Street, Kingston, the dispensary beingsituated in Paterson Street, and this last department will still be continued. The new hospital is a handsome building, and consists of two blocks, connected by corridors. The hospital block, oft wo floors, contains a ward for ten beds and two small wards on each. In the administration block the operating theatre and lecture rooms are placed, and the nurses are housed here also. The total cost is estimated at £12,000.

What the future of the so-called serum treatment will be it is difficult to foretell. Whether the outcome of the investigations which are being carried on will be to simplify matters and to discover among the various anti-toxins a common factor which will be efficient against all toxins, a thing not altogether outside the range of legitimate aspirations, or whether the drift of discovery will be towards the more complete isolation of the individual serums which are opposed to individual conditions, is a point of great philosophical importance, but is of interest in regard to the future rather than to the practitioners of to-day. For the present we have to depend on such serums as are placed on the market, and it must be confessed that medical men are in a condition of peculiar helplessness in the matter; for beyond taking care that they purchase a good "brand" they have to trust entirely to the skill and honesty of the trade, there being no government inspection or control over such affairs in England. In France, however, the semi-official position and the high scientific repute of the Pasteur Institute (for whom Messrs. Oppenheimer and Co. are the wholesale agents in this country), to some extent removes the difficulty, as practitioners are able to obtain from that establishment serums of known efficacy and of standard strength.

